# Bronchiolar adenoma/ciliated muconodular papillary tumor complicated by lymphoid interstitial pneumonia in a patient with Sjögren's disease: A case report and systematic review

**DOI:** 10.1111/1759-7714.15420

**Published:** 2024-08-18

**Authors:** Pinar Çağan, Ali Kimiaei, Seyedehtina Safaei, Houssam Eddine Youcefi, Alara Abu Saadeh, Feride Yaman, Özlem Yapıcıer, Cemal Asim Kutlu

**Affiliations:** ^1^ Department of Thoracic Surgery Bahçeşehir University Istanbul Turkey; ^2^ Department of Pulmonology Bahçeşehir University Istanbul Turkey; ^3^ Department of Pathology Bahçeşehir University School of Medicine, Göztepe Medical Park Training and Education Hospital Istanbul Turkey

**Keywords:** ciliated muconodular papillary tumor, CMPT, lung tumor, lymphoid interstitial pneumonia, Sjögren's disease

## Abstract

Bronchiolar adenoma (BA)/ciliated muconodular papillary tumor (CMPT) is a rare pulmonary neoplasm, with less than 150 cases documented in the literature. We report a unique case of BA/CMPT complicated by lymphoid interstitial pneumonia (LIP) in a 55‐year‐old male with Sjögren's disease. This is the first documented instance of such a comorbidity. Through a systematic review of PubMed, we also summarize the demographic, clinical, radiological, histopathological, and treatment characteristics of CMPT.

## Graphical abstract

Bronchiolar adenoma (BA)/ciliated muconodular papillary tumor (CMPT) is a rare pulmonary neoplasm, with less than 150 cases documented in the literature. We report a unique case of BA/CMPT complicated by lymphoid interstitial pneumonia (LIP) in a 55‐year‐old male with Sjögren's disease. This is the first documented instance of such a comorbidity. Through a systematic review of PubMed, we also summarized the demographic, clinical, radiological, histopathological, and treatment characteristics of CMPT.

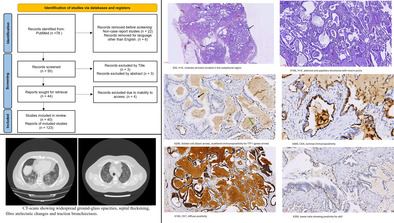

## INTRODUCTION

First described by Ishikawa in 2002,^1^ ciliated muconodular papillary tumor (CMPT) of the lung is a newly defined rare tumor with less than 150 cases reported in the literature. He named this tumor based on its morphological traits, including ciliated columnar cells, goblet cells, and basal cells.^1^ In 2018, Chang et al. designated these lesions as bronchiolar adenomas (BAs) because the morphological and immunohistochemical features ranged from those resembling proximal bronchioles to those resembling respiratory bronchioles.^2^ In 2021, BA/CMPT was defined as a subtype of epithelial tumor adenoma in the fifth edition of the WHO Lung Tumor Classification.^3^ CMPT predominantly affects East Asian populations, especially the elderly.^4^, ^5^ The rarity of this tumor and the lack of knowledge regarding its diagnosis and treatment pose a challenge for physicians. Here, we systematically review the literature and introduce a new case of CMPT. To our knowledge, this is the first case of CMPT complicated by lymphoid interstitial pneumonia in a patient with Sjögren disease.

## METHODS

The current study is a systematic review of case reports of ciliated muconodular papillary tumors, and it also includes a unique case report. This study was conducted in March 2024 to review all the published case reports in PubMed. The search was conducted on PubMed by combining Boolean operators with the following key words: “ciliated muconodular papillary tumor,” “CMPT,” “case report,” and “case series.” First, the reviewers screened titles based on the objectives of the study. Subsequently, the abstracts were reviewed. The related abstracts for our study were selected, and full copies of the articles were extracted from the literature and used for this study.

The inclusion criteria were related articles or literature published in English, including keywords in their titles, and a confirmed diagnosis of CMPT. We found 72 articles. Articles that did not meet the inclusion criteria, such as those not written in English, lacking a confirmed diagnosis of CMPT, inability to retrieve data, or not being case reports or case series, were excluded. Two authors separately read and reviewed the full texts of the retrieved articles to identify various aspects of CMPT, including patient demographics such as age and sex, smoking history, tumor size and location, radiological and histological findings, genetic mutations, treatment modalities, and outcomes.

Patient consent was obtained for the inclusion of clinical data and any accompanying images in this study.

## RESULTS

**FIGURE 1:**
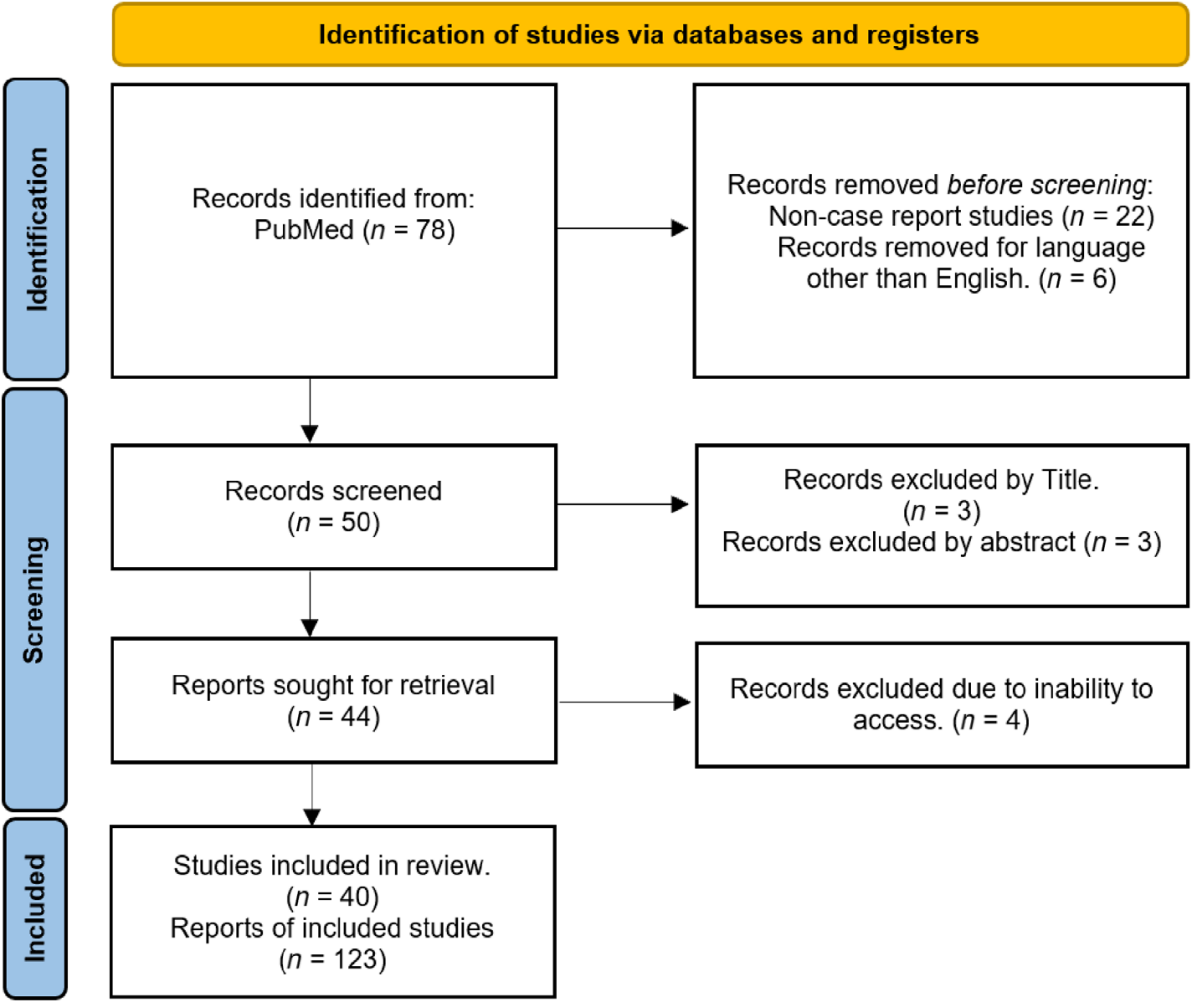
PRISMA diagram illustrating the flow of study selection in the systematic review.

A total of 50 studies covering 123 cases were included in this review. The selection process is shown in Figure 1.

The clinical data of our patient and the 123 cases reported in the literature to date are summarized in Table 1.

**TABLE 1:** Summary of clinical data on the cases reported in the literature.

Year	Source	No of cases	Age and sex	Smoking history	Tumor size (mm)	Tumor location	Radiological findings	Histological findings	Gene mutation	Treatment	Outcome: NED (months)
2020	Abe et al^6^	1	76/male	YES	14	RLL	Irregular nodule	Papillary tumor with central fibrosis, proliferating along the alveolar walls, surrounded by mucous lakes, and consisting of ciliated columnar cells and goblet cells with basaloid cell proliferation	Unknown	Segmentectomy	14
2018	Chang et al^2^	21	Mean: 75.1 (range: 55–78) M:F = 11:10	YES: 15 NO: 4 Unknown: 2	7.7 range: (2–20)	Unknown	Peripheral solid lesion: 8 Ground class: 4 Mixed solid/ground glass: 2 Unknown: 7	Unknown	BRAF V600E: 8 EGFR: 4 KRAS: 4 HRAS: 1 Unknown: 4	Unknown	11 range (1–9)
2017	Chu et al^7^	1	56/Male	Unknown	8	LUL	Irregular poorly enhanced nodule	Endo bronchiolar and peribronchiolar papillary growing in a central cyst consisting of different cells, including ciliated columnar, goblet, and small cells	Unknown	Segmentectomy	5
2014	Chuang et al^8^	1	68/male	YES	12	RLL	Solitary ground glass nodule	Nodular papillary composed of ciliated cells and nonciliated columnar cells, goblet cells, and basaloid cells in a mucin pool	Unknown	Wedge resection	48
2022	Coşgun et al^9^	1	50/Male	YES	10	RLL	Cavitary lung lesion	Mucinous and nonmucinous ciliated cells with minimal cellular atypia	Unknown	Lobectomy	Unknown
2022	Du et al^10^	2	63/Female 58/Female	Unknown	6 12	RLL RUL	Subpleural nodules Solid lobulated nodule	Atypical cells, visible cilia, and bilayer structures in focal areas. Local cells had atypia, and cilia were found locally.	Unknown HER2	Wedge resection	11 12
2008	Harada et al^11^	1	62/ Male	YES	9	LLL	Irregularly shaped nodule	Papillary tumor composed of ciliated columnar and goblet cells surrounding by a mucous nodule	Unknown	Partial resection	24
2013	Hata et al^12^	1	76/Female	NO	7 mm	LUL	Irregular nodule	Papillary tumor with central fibrosis, proliferating along the alveolar walls, surrounded by mucous lakes, and consisting of ciliated columnar cells and goblet cells with basaloid cell proliferation	Unknown	Lobectomy	23 months
2016	Ishikawa et al^13^	5	Mean: 72.4 (66–82) M:F = 3:2	YES: 3 NO: 2	Mean: 50 (Range:5–45)	RUL: 1 RLL: 2 LLL: 2	Peripheral nodule: 3 Peripheral ground glass opacities: 1 Irregular consolidation‐like lesion: 1	Ciliated columnar cells with mucous lakes.	Unknown: 5	Lobectomy: 2 Partial resection: 3	Mean: 41.6 (range: 19–58)
2017	Jin et al^14^	1	59/ Female	NO	8	RLL	Peripheral part solid nodule exhibiting central cavity	Nodular papillary tumor composed of proliferating epithelial cells and peripheral abundant extracellular mucin	Unknown	Lobectomy	6 months
2015	Kamata et al^15^	10	Mean: 62 (range: 56–78) M:F = 7:3	YES: 5 NO: 5	Mean: 10 (range: 6–15)	RUL: 1 LLL: 4 RLL: 5	Small peripheral solid or part‐solid nodules with an irregular contour	Predominantly glandular: 5 Predominantly papillary: 5	BRAF G606R: 2 BRAF V600E: 3 EGFR: 3	Wedge resection: 8 Lobectomy: 1 Segmentectomy: 1	Mean: 42.8 (range: 2–88)
2019	Kashima et al^16^	5	Mean: 67.2 (range: 56–73) M:F = 2:3	Unknown	Mean: 11.2 (range: 8–18)	RLL: 2 LLL: 2 LUL: 1	Unknown	All tumors consisted ciliated columnar cells, mucinous cells, and basal cells arranged in papillary and glandular structure	BRAF V600E: 3	Unknown	Mean: 34.8 (range: 6–72)
2017	Kim et al^17^	1	72/male	YES	9	LLL	Ground glass nodule	Adenomatous proliferation and focal broad papillary fronds of bland‐looking epithelial cells The luminal surface of papillary fronds and some glands were composed of organized growth of three cellular components including mucous cells, ciliated columnar cells and underlying basal cells	BRAF V600E	Wedge resection	36
2016	Kon et al^18^	5	Mean: 71.2 (range: 66–80) M:F = 3:2	Unknown	Mean: 9.4 (range: 7–13)	LLL: 1 RLL: 3 LUL: 1	Irregular border	Unknown	Unknown	Wedge resection: 4 Lobectomy: 1	Mean: 24.2 (range: 5–48)
2015	Lau et al^19^	1	19/ Female	NO	12	RLL	Eccentric lucent area suggesting cavitation	Ciliated tumor cells and goblet cells	Unknown	Wedge resection	Unknown
2023	Liao et al^20^	1	38/ female	Unknown	13	RML	Cavitary nodule	The cavities were lined purely by mucinous luminal cells, and a continuous layer of basal cells was under the mucinous luminal cells	Unknown	Lobectomy	24
2016	Liu et al^21^	4	Mean: 74 (range: 60–83) M:F = 1:3	YES: 1 Unknown: 3	Mean: 8 (range: 4–12)	RLL: 1 RML: 1 LLL: 1 LUL: 1	Peripheral nodule	Unknown	BRAF V600: 1 AKT1 E17K: 1	Wedge resection: 3 Lobectomy: 1	7: 1 120: 1 Unknown: 2
2023	Liu et al^22^	3	Mean: 63 (range: 49–75) M:F = 0:3	NO: 2 Unknown: 1	Mean: 15 (range: 7–24)	LLL: 2 RML: 1	Mixed ground glass nodules	Ciliated cells, mucus cells, and basal cells	Unknown: 1 BRAF V600: 1 EGFR 19‐del: 1	Wedge resection: 2 Lobectomy: 1	Unknown
2023	Liu et al^23^	1	57 Female	NO	17	LLL	Mixed solid and ground‐glass nodule	Comprised mainly of basal cells and luminal epithelial cells. The luminal layer was predominantly formed of mucinous, ciliated columnar, and cuboidal or low columnar cells in various numbers and proportions	Unknown	Wedge resection	Unknown
2020	Matsushima et al^24^	1	60/ Male	Unknown	10	LLL	Ground‐grass nodule	Proliferated epithelium surrounding alveoli including ciliated columnar, mucous and basal cells	Unknown	Wedge resection	20
2019	Mikubo et al^25^	1	69/ Male	NO	13	LLL	Solid nodule with central cavitation	Abundant ciliated columnar cells and mucous cells arranged in sheets and clusters	Unknown	Wedge resection	8
2018	Miyai et al^26^	1	67/Female	YES	18	RML	High density nodule with ground glass opacities	Mixture of ciliated columnar cells, mucous cells and basal cells	Unknown	Partial resection	4
2022	Moon et al^27^	1	39/Male	Unknown	10	RLL	Solid nodule with thin cavitary wall	Mixture of ciliated columnar and basal cells with papillary structures interspersed between the mucous lakes	Unknown	Lobectomy	15
2020	Murakami et al^28^	1	70/ female	YES	5	RLL	Cavitary lesion	Proliferating papillary cells with cilia adjacent to the bronchi and mucous glands around the cystic wall	Unknown	Wedge resection	7
2020	Onishi et al^29^	15	Median: 67 (range: 60–80) M: F = 8:7	Unknown	Mean: 9 (range: 6–14)	RLL: 8 LLL: 4 LUL: 2 RML: 1	Unknown	All tumors composed of cellular and mucinous components	Unknown	Unknown	Unknown
2021	Patané et al^30^	1	68/Female	YES	18	RLL	Nodule with irregular border	Focal area of irregular fibrosis and a papillary neoplasm with ciliated columnar epithelial cells, mucin producing cells, basal cells, mucus lakes within the alveoli and absence of necrosis and nuclear atypia	Unknown	Lobectomy	Unknown
2010	Sato et al^31^	2	67/Male 59/Female	YES: 1 NO: 1	9 7	RUL: 1 LLL: 1	Ground‐glass opacity Central cavity	Papillary tumors with a mixture of ciliated epithelial and goblet cells	Unknown	Partial resection	10 18
2019	Shao et al^32^	2	58/Female 66 Female	Unknown	8 6	LLL RLL	Ground‐glass nodule	Tumor cells arrayed irregularly along the alveolar wall and were tubular. Arrayed tubular structures, the mucus was visible in the lumen, and micropapillary tumor cells were found in the mucus.	KRAS‐G12D and KRAS‐G12A BRAF‐V600E	Wedge resection	Unknown
2019	Shen et al^33^	2	58/ Male 64/ Female	Unknown	11 8.5	RLL LLL	Solitary nodule with central cavity	Proliferated epithelium with adenoid and papillary structures, including ciliated columnar, mucous and basal cells. Ciliated columnar cells with mucous lakes.	Unknown	Lobectomy Wedge resection	Unknown
2021	Shirsat et al^34^	18	Median: 72 (range: 56–83) M:F = 8:10	YES: 12 NO: 4 Unknown: 2	Median: 9 (range: 2–20)	RLL: 8 RUL: 1 RML: 3 LLL: 2 LUL: 3 Unknown: 1	Mass lesion: 12 Concomitant carcinoma: 4 Unknown: 2	Unknown	Unknown	Unknown	Unknown
2023	Sun et al^35^	1	55/ Male	YES	1 10 mm nodule and multiple small nodules (3‐7 mm)	LLL	Irregular borders and small ridges on the edge, accompanied by a vacuole sign	Papillary hyperplasia of the bronchial epithelium of, rich in mucous cells, similar to a single‐layered cell structure with atypical manifestations and another lesion was bronchial epithelial hyperplasia with a double‐layered cell structure. Another lesion showed bronchial epithelial hyperplasia with a double‐layered cellular structure, visible mucous cells, and atypical focal epithelial cells.	Unknown	Segmentectomy and wedge resection	37
2017	Taguchi et al^36^	1	84/ Female	NO	8	RLL	Well‐circumscribed nodule	Proliferation of a mixture of ciliated, goblet and basal cells	Unknown	Partial resection	10
2019	Uchida et al^37^	1	78/ Male	Unknown	19	RUL	Nodule with cavity	Mixture of ciliated columnar, mucous, and basal cells in glandular and papillary growth patterns	Unknown	Lobectomy	30
2023	Uchiyama et al^38^	1	68/ Female	Unknown	13	RUL	Part solid nodule	Papillary growth and Bilayering with basal cells was observed without disturbance of the arrangement of the basal cells. The luminal side showed epithelial columnar cells and goblet cells, which secreted mucous material	Unknown	Partial resection	Unknown
2017	Udo et al^39^	4	Median: 67 M:F = 0:4	NO	Median: 11 (range: 8–25)	Unknown	Unknown	The tumors typically exhibited a mixture of acinar and papillary growth patterns without clear evidence of invasion, and were surrounded by abundant mucus pools in the alveolar spaces. The tumors were composed of two basal cell layers and surface epithelia. The latter consisted of an uneven mosaic of ciliated columnar, goblet, and mucin‐producing epithelial cells of the gastric type.	BRAF V600E: 1 KRAS G12D: 1 AKT1 E17K: 1	Lobectomy: 3; Segmentectomy:1	Unknown
2021	Wang et al^40^	1	79/ Male	NO	30	RUL	Solid tumor with irregular borders	Tubular and papillary structures. Mixture of proliferating ciliated, basal cells and mucous cells	EGFR	Wedge resection	24
2021	Wang et al^4^	1	64/ Female	Unknown	12	RLL	Solid nodule invading the pleura with ill‐defined margins	Ciliated columnar cells were coated on the surface of the adenoid or papillary structure. The tumor cells showed no necrosis, atypia or mitosis and presented as a mixture of bland ciliated columnar cells, a basal cell layer and mucinous cells.	Unknown	Wedge resection	Unknown
2019	Yao et al^41^	1	67/ Female	Unknown	12	LUL	Irregular solid nodule with a maximum diameter	Proliferated epithelium with adenoid and papillary structures, including ciliated columnar cells, mucous cells, and basal cells. Ciliated columnar cells were coated on the surface of adenoid or papillary structures. Basal cells were located in the outer layer	Unknown	Segmentectomy	10
2021	Zhao et al^42^	1	53/ Male	Yes	NA	RLL	Bilateral lung interstitial inflammation with edema (no nodule)	Abundant basal cells, ciliated columnar epithelial cells, and mucous cells in disorganized manner	Unknown	Watch and wait	Unknown

Abbreviations: LLL, left lower lobe; LML, left middle lobe; LUL, left upper lobe; NED, no evidence of disease; RLL, right lower lobe; RML, right middle lobe; RUL, right upper lobe.

Among the reported cases, 59 were male and 65 were female, indicating a relatively balanced distribution of cases between sexes. The age range of the patients varied from 19 to 84 years, reflecting the occurrence of CMPT across a wide age spectrum. Regarding smoking history, data were available for 77 patients, with 48 reported as smokers and 29 as nonsmokers. However, smoking status remained unknown in 47 individuals, indicating a significant proportion of missing data in this regard. The tumor size ranged from 2 to 45 mm, demonstrating variability in the dimensions of the CMPT lesions among the patient cohort. The analysis revealed a notable predominance of CMPT on the right side of the lung, with 60 cases identified compared to 38 cases on the left side. Tumors occurred more frequently in the lower lobes of the lungs than in the upper lobes. Specifically, the right lower lobe (RLL) emerged as the most common site of presentation, with 44 cases identified, surpassing the occurrences in other lung lobes. Regarding treatment modalities, the data delineated various approaches adopted for managing ciliated muconodular papillary tumors. Radiological findings encompassed a variety of presentations, including irregular nodules, ground glass nodules, and cavitary nodules. Hence, the radiological findings were nonspecific, necessitating confirmation of the diagnosis through histological examination. Histological examination revealed the characteristic features of ciliated muconodular papillary tumors, including papillary structures surrounded by mucous lakes. The tumor tissue exhibited a composition of ciliated and columnar cells, along with goblet and basal cell proliferation, consistent with the histopathological profile of CMPT. Gene mutation analyses revealed several mutations in the study cohort. Specifically, *BRAFV600E* mutations were observed in 11 cases, followed by *EGFR* mutations in five patients. Additionally, *KRAS* G12D mutations were found in two patients, as were *HER2* mutations. *BRAF* G606R mutations were detected in two cases, and *AKT1* E17K mutations were observed in two patients. Regarding treatment modalities, the data delineated various approaches adopted for managing ciliated muconodular papillary tumors. Among the documented cases, one patient underwent segmentectomy, 18 patients underwent lobectomy, 32 patients underwent wedge resection, and nine patients underwent partial resection. However, treatment specifics remained unmentioned in 38 cases, highlighting gaps in the information regarding the management strategies employed in these instances. No evidence of disease recurrence was reported within the follow‐up period of up to 120 months.

## CASE REPORT

### Patient information

**FIGURE 2:**
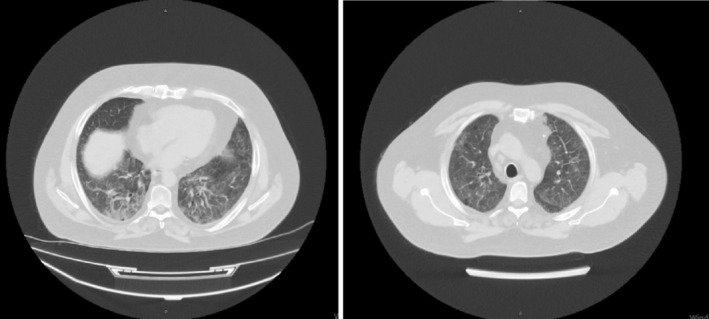
Computed tomography scans showing widespread ground‐glass opacities, septal thickening, fibro atelectatic changes and traction bronchiectasis.

A 55‐year‐old male with a history of Sjögren's syndrome diagnosed 8 months prior and currently on steroid therapy (methylprednisolone 0.75 mg/kg) presented to our clinic on February 13, 2024 with a one‐week history of cough and shortness of breath. Steroid dosage at the first consultation was 0.5 mg/kg. He had a past medical history significant for triple coronary artery bypass grafting (CABGx3) 13 years ago. Prior imaging on January 2, 2024, revealed widespread ground‐glass opacities in both lungs, with more pronounced involvement in the lower zones. Subsequent computed tomography (CT) imaging on January 30, 2024 corroborated these findings, demonstrating widespread ground‐glass opacities, septal thickening in both lungs. Additionally, fibroatelectatic changes and traction bronchiectasis were noted in both lower lung lobes (Figure 2).

### Clinical findings

Upon physical examination, the patient was pain‐free, rales were detected in the respiratory sounds, with no other notable findings observed. Subsequently, the patient's steroid treatment was ceased. Given the disparities between reports and clinical assessment, a recommendation was made for an open lung biopsy.

### Diagnostic assessment

#### Biopsy

The patient underwent a right lung biopsy, with wedge resections performed in two localizations: right upper lobe (dimensions: 3 × 2.5 × 0.6 cm) and right lower lobe (dimensions: 6.5 × 2 × 0.5 cm). A 20 F drain was placed postoperatively. The patient was discharged on February 14, 2024 for outpatient follow‐up. We decided to do a salivary gland biopsy due to uncertainty of the Sjogren diagnosis and the results were consistent with Sjögren's syndrome.

#### Histopathological findings

**FIGURE 3:**
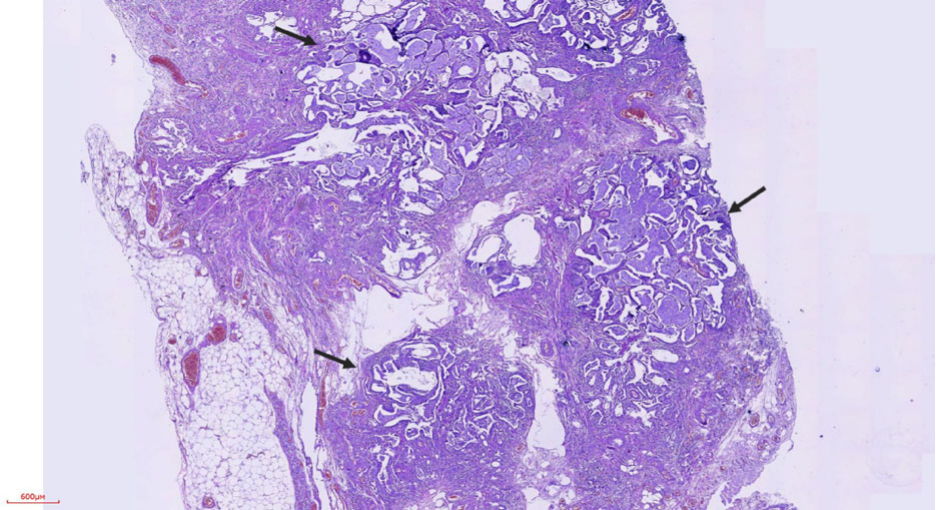
X20, hematoxylin and eosin, nodules (arrows) located in the subpleural region.

**FIGURE 4:**
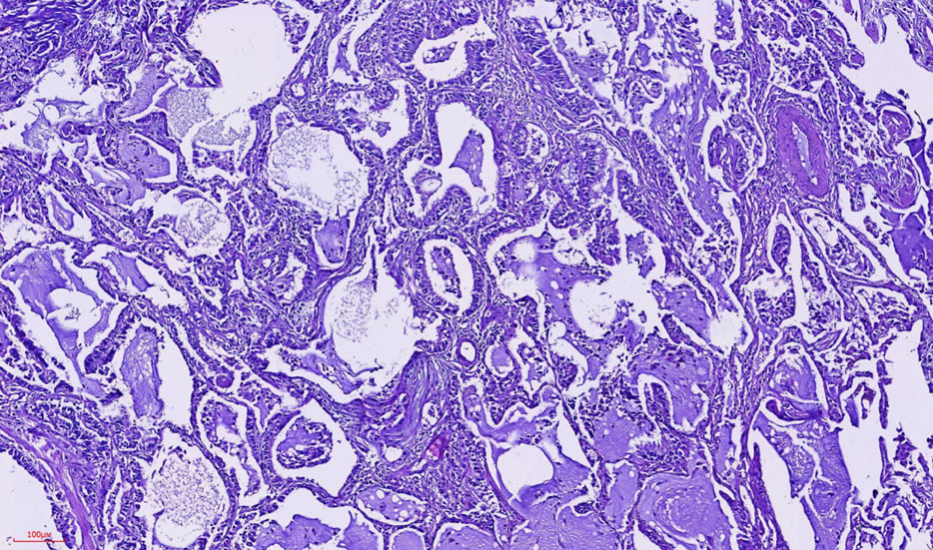
X100, hematoxylin and eosin, adenoid and papillary structures with mucin pools.

**FIGURE 5:**
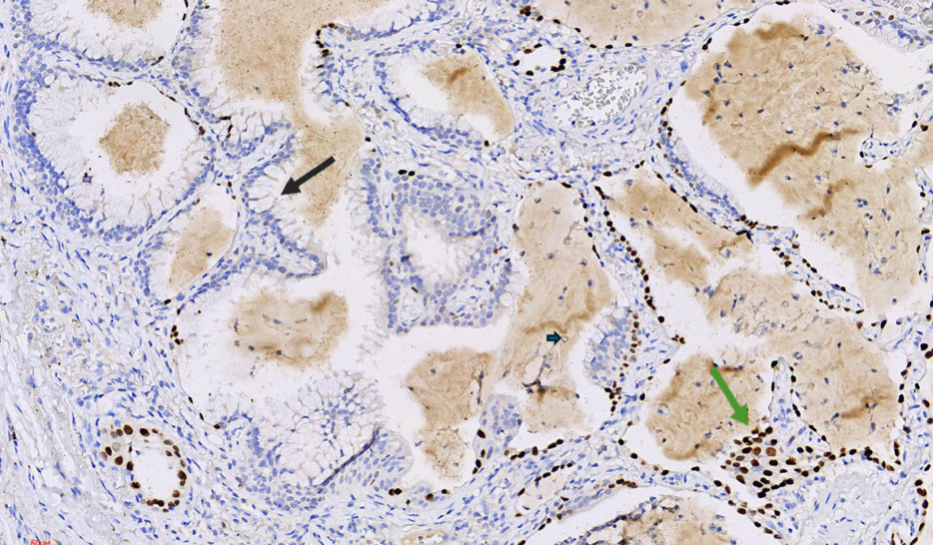
X200, Goblet cell (black arrow), scattered immunopositivity for TTF1 (green arrow).

Histopathological examination of the biopsy specimens revealed fibrosis and diffuse mature lymphocytic infiltration, consistent with lymphoid interstitial pneumonia (LIP). Additionally, in the right upper lobe sample, nodular lesions were identified, primarily situated in the subpleural region and displaying multifocal distribution (Figure 3). These nodules exhibited adenoid and papillary structures (Figure 4), comprising three distinct cell types: ciliated columnar cells, mucinous Goblet cells (Figure 5, black arrow), and basal cells, with the largest measuring up to 5 mm in diameter. No epithelial atypia or mitotic activity was observed. These features are in line with the diagnosis of CMPT localized in the subpleural area of the right lower lobe.  

#### Immunohistochemical analysis

**FIGURE 6:**
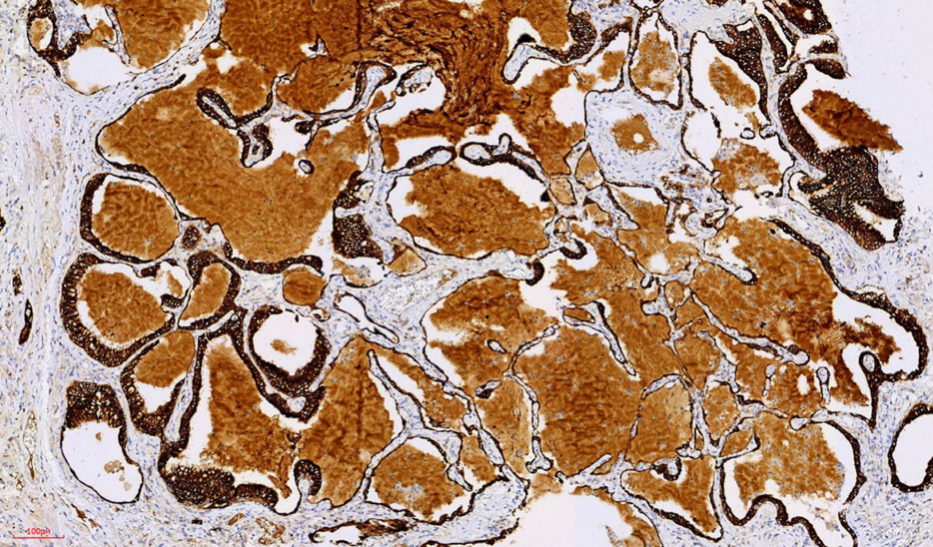
X100, CK7, diffuse positivity.

**FIGURE 7:**
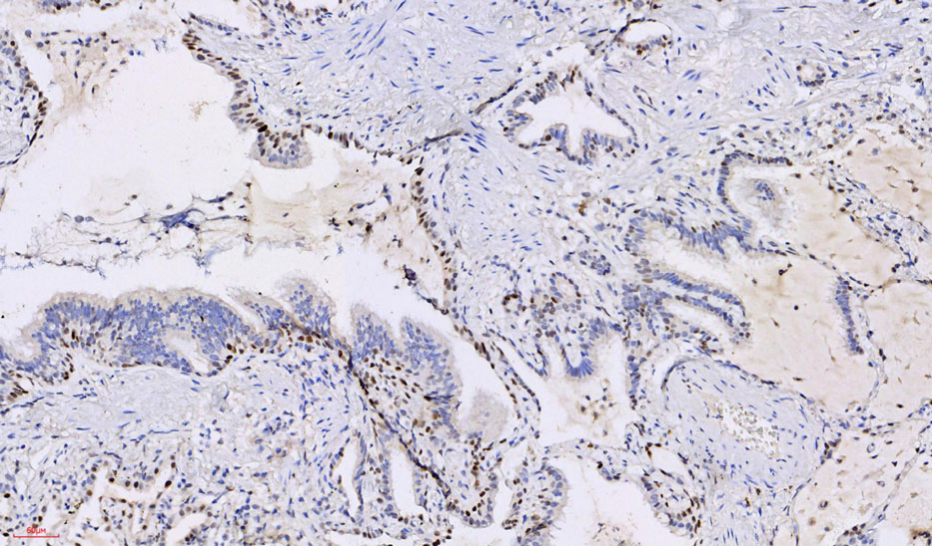
X200, basal cells showing positivity for p63.

**FIGURE 8:**
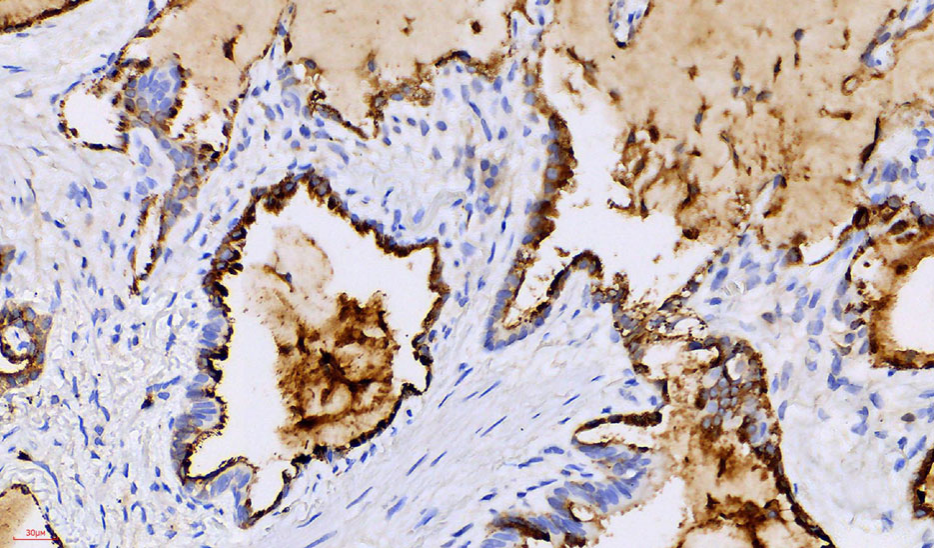
X400, carcinoembryonic antigen (CEA), luminal immunopositivity.

Immunohistochemical staining showed diffuse positivity for, CK7 (Figure 6), scattered positivity for TTF1 (Figure 5, green arrow), basal positivity for P63 (Figure 7), and luminal positivity for carcinoembryonic antigen (CEA) (Figure 8), supporting the diagnosis of CMPT.

### Therapeutic intervention

The patient did not have a solid resectable lesion identifiable on CT scan; therefore, surgery was not performed. Subsequently, treatment with steroids (methylprednisolone 1 mg/kg) and Cellcept (mycophenolic acid 500 mg twice daily) was initiated.

## DISCUSSION

This study investigated the epidemiological, clinical, and molecular characteristics of ciliated muconodular papillary tumors (CMPT). Here, we discuss the findings of our study under the following main headings:

### Demographic characteristics and distribution of cases

The systematic review revealed a relatively balanced distribution of CMPT cases between males and females. While the age range of the patients varied from 19 to 84 years, the tumor almost always occurred in older adults, with only one occurrence of BA/CMPT in adolescents.^19^ Regarding smoking history, data were available for 77 patients, with 48 reported as smokers and 29 as nonsmokers. However, the smoking status remained unknown in 47 individuals, indicating a significant proportion of missing data in this regard. Our patient was a 55‐year‐old male patient with no history of smoking. Overall, these findings suggest that BA/CMPT tends to affect older adults, occurs relatively equally between males and females, and that there is an incomplete understanding of its association with smoking due to missing data in a significant proportion of cases.

### Clinical presentation and radiological findings

While we did not include the clinical presentation of BA/CMPT in our study, the literature suggests that the majority of cases are asymptomatic, and even in clinically symptomatic patients, symptoms are mostly nonspecific manifestations such as cough and sputum.^22^ Given that, the tumor was mostly found incidentally on CT imaging. Although our patient presented with cough and dyspnea, it could also be a result of LIP.

Referring to the literature, the radiological findings comprise a range of presentations, encompassing irregular nodules, ground glass nodules, and cavitary nodules. There is only one case report in which chest CT showed bilateral interstitial lung inflammation with edema.^42^ Widespread bilateral ground‐glass opacities, with more pronounced involvement in the lower zones, septal thickening, fibro‐atelectatic changes, and traction bronchiectasis in the lower lung lobes in our patient, are similar to this case. In both of these cases, there was no resectable tumor, which makes the management of these scenarios challenging.

### Tumor characteristics and location

The tumor sizes varied significantly within the patient cohort, ranging from 2 to 45 mm, showing diversity in the dimensions of the CMPT lesions. Examination of the data revealed a distinct prevalence of CMPT on the right lung side. Moreover, tumors were more prevalent in the lower lobes of the lungs than in the upper lobes. Notably, the right lower lobe (RLL) emerged as the predominant site of presentation. In the biopsy specimen of our patient, nodular lesions that were not visible on CT imaging were identified as CMPT in the right upper lobe sample.

### Histological findings

BA/CMPT is distinguished by the formation of nodular growths along the alveolar lung parenchyma, composed of a benign bilayered epithelium reminiscent of bronchioles. This epithelium comprises a continuous layer of basal cells surrounding the luminal cells. Luminal cells exhibit varying degrees of differentiation; some resemble proximal airways, characterized by abundant ciliated and/or mucinous cells, whereas others resemble distal airways, predominantly cuboidal, TTF‐1‐positive cells with sparse or absent ciliated and mucinous cells.^3^ In our patient, the nodules exhibited adenoid and papillary structures, comprising three distinct cell types: ciliated columnar cells, mucinous Goblet cells, and basal cells, with the largest measuring up to 5 mm in diameter. Epithelial atypia, or mitotic activity, was not observed. These features are consistent with the diagnosis of a CMPT localized in the subpleural area of the right lower lobe.

### Correlation between CT imaging patterns and pathological findings

Table 2 illustrates the correlation between various CT imaging patterns and their corresponding pathological findings in bronchiolar adenoma (BA)/ciliated muconodular papillary tumor (CMPT). CT patterns can be broadly classified into five categories: solid nodules, ground glass nodules (GGNs), mixed solid and ground glass nodules, cavitary lesions, and irregular and lobulated nodules. Solid nodules are associated with papillary tumors composed of ciliated columnar cells, mucous cells, and basal cells arranged in papillary and glandular structures. GGNs correspond to adenomatous proliferation with focal broad papillary fronds, including mucous cells, ciliated columnar cells, and basal cells. Mixed nodules exhibit a combination of basal cells and luminal epithelial cells, with mucinous and ciliated columnar cells. Cavitary lesions are lined by mucinous luminal cells and a continuous layer of basal cells, showing minimal cellular atypia. Irregular and lobulated nodules reflect papillary tumors with complex cellular compositions, including ciliated columnar cells, goblet cells, and mucous nodules.

**TABLE 2:** Correlation between computed tomography (CT) imaging patterns and pathological findings in ciliated muconodular papillary tumor.

Broad CT pattern classification	Pathological findings
Solid nodules	Papillary tumors, nodules with ciliated columnar cells, mucous cells, and basal cells arranged in papillary and glandular structures
Ground‐glass nodules (GGNs)	Adenomatous proliferation with focal broad papillary fronds, including mucous cells, ciliated columnar cells, and basal cells
Mixed solid and ground glass nodules	Combination of basal cells and luminal epithelial cells, with mucinous and ciliated columnar cells
Cavitary lesions	Lined by mucinous luminal cells and a continuous layer of basal cells, showing minimal cellular atypia
Irregular and lobulated nodules	Papillary tumors with complex cellular compositions, including ciliated columnar cells, goblet cells, and mucous nodules

### Genetic mutations and molecular profiling

Gene mutation analyses revealed several mutations in the study cohort. Specifically, *BRAF* V600E mutations were observed in 11 cases, followed by *EGFR* mutations in five patients. Additionally, *KRAS* G12D mutations were found in two patients, as were *HER2* mutations. *BRAF* G606R mutations were detected in two cases, and *AKT1* E17K mutations were observed in two patients. Data on gene mutations in the remaining cases remain unknown. Gene mutation analysis was not performed in our case.

### Treatment approaches and management strategies

Surgical resection remains the primary treatment for CMPT, with lobectomy being the most common approach. However, in one patient, owing to the absence of a solid, resectable lesion identifiable on a CT scan, a strategy of waiting and watching was applied.^42^ We also adopt this strategy. Treatment specifics remained unmentioned in 38 cases, highlighting gaps in information regarding the management strategies employed in these instances.

### Growth rate, prognosis, and outcomes

The growth rate of BA/CMPT is approximately 0.49 mm per year, aligning with the observed growth rates of benign lung tumors, which range from 0.5 to 5 mm per year.^27^, ^29^, ^43^ Surgical intervention has been highly effective, with all patients responding positively and no evidence of disease recurrence observed during follow‐up periods extending up to 120 months.

### Differential diagnosis

A summary of the characteristics and distinguishing features of the differential diagnoses of BA/CMPT is presented in Table 3.

**TABLE 3:** Differential diagnosis of BA/CMPT.

Differential diagnosis	Characteristics	Differentiating features from BA/CMPT
Invasive mucinous adenocarcinoma	‐ Gland cavity or nipple structure—mainly mucus secretion of columnar cells—rarely cilia or immature cilia structure—presence of mucus‐filled gland cavities—small tumor cells with less atypia—absence of complete basal cells	Lack of complete basal cells
Low‐grade mucoepidermoid carcinoma	‐ Large amounts of mucus components—mainly composed of mucus cells, epidermoid cells, and intermediate cells—occurs in main bronchus of young people—CD117 positive on immunohistochemistry	Absence of complete basal cells; Lack of papillary structure; CD117 positive
Acinar type adenocarcinoma	‐ Predominantly acinar structure—no mucus in the cavity—tumor cells with atypia—absence of complete basal cells—continuity with normal bronchioles in distal cases	Absence of complete basal cells; presence of acinar structure
In situ adenocarcinoma	‐ Less atypia with adherent growth mode—mainly tubular glands with cubic or low columnar cells—often misdiagnosed in frozen sections	Similar adherent growth mode; immunohistochemical labeling of basal cells for differentiation

*Note*: References ^6^, ^22^, ^44^, ^45^.

Abbreviations: BA, bronchiolar adenoma; CMPT, ciliated muconodular papillary tumor.

### BA/CMPT and LIP

LIP is a rare benign pulmonary lymphoproliferative disorder characterized by diffuse involvement of the lung parenchyma by reactive pulmonary lymphoid tissue.^46^ It is often associated with autoimmune disorders or infections.^47^ The two main conditions associated with LIP are Sjögren syndrome for autoimmune diseases and human immunodeficiency virus (HIV) for infections.^47^, ^48^, ^49^, ^50^ The clinical presentation of LIP is classically characterized by an insidious onset with exertional dyspnea and nonproductive cough, and in some cases associated with general symptoms including fever, night sweats, and weight loss.^47^, ^51^, ^52^, ^53^ LIP is usually suspected in the patient with an associated rheumatic disorder (e.g., Sjögren's disease) and characteristic radiographic findings.^50^ LIP shows diverse radiographic patterns, including basilar reticular opacities or nodular densities. As it advances, a mix of ground‐glass and consolidative opacities develops, with air bronchograms visible in larger lesions. Nodular disease is more frequent in HIV patients.^51^, ^54^, ^55^ However, the definitive diagnosis is lung biopsy that demonstrates extensive alveolar septal infiltration with lymphocytes, plasma cells, and histiocytes.^56^, ^57^ While there is no specific treatment protocol universally established for LIP, in cases where LIP is associated with autoimmune diseases, treatment may include a combination of systemic corticosteroids and cytotoxic drugs, which have shown success in many cases.^58^ While approximately 5% of cases of LIP transform to lymphoma it is unclear whether LIP undergoes malignant transformation or whether the lymphoma develops as a comorbid process.^48^ The cystic lesions observed in LIP result from the expansion of alveolar structures and do not exhibit the distinct cellular characteristics of CMPT. Similarly, the solid nodules in LIP, often related to amyloid deposits or calcifications, differ histopathologically from CMPT lesions. The presence of multifocal BA/CMPT observed in this case may be associated with LIP and underlying Sjögren's disease in the patient; however, this association remains speculative. It is important to note that CMPT is primarily a neoplastic lesion with identifiable molecular characteristics and potential for malignant transformation, and thus, it is important to note that CMPT is of a neoplastic nature, distinct from the secondary changes that may be observed in LIP. Through differential diagnosis is crucial to distinguish these lesions from the reactive changes seen in LIP.

This study had several limitations that warrant consideration. First, the search was confined to PubMed, excluding other databases, which may have resulted in missing relevant studies. Second, our systematic review focused exclusively on CMPT and did not encompass BA in the search strategy. Consequently, studies solely reporting BA in their text might have been inadvertently omitted, potentially leading to an incomplete representation of pertinent literature. Third, the rarity of CMPT presents a challenge in accruing a sizable sample size, thereby constraining the depth of our analysis and the precision of our conclusions. Future investigations could benefit from collaborative endeavors spanning multiple institutions to assemble a larger cohort of CMPT cases, facilitating more comprehensive analyses of demographic, clinical, and molecular characteristics. Furthermore, prospective studies featuring extended follow‐up periods are warranted to delineate the long‐term outcomes, recurrence rates, and treatment responses of CMPT, thereby augmenting our understanding of this rare entity and informing clinical management strategies.

In conclusion, BA/CMPT of the lung is an extremely rare tumor characterized by a tripartite cellular composition with ciliated columnar cells, mucinous cells, and basal cells forming a papillary structure with mucin pools. These distinct histological features aid in the diagnosis and differentiation of CMPTs from other pulmonary neoplasms. Presenting as a nodular lesion is not always the case, and the tumor can also present as interstitial lung inflammation, for which choosing surgery as treatment is unsuitable. Comorbidities such as LIP and Sjogren's disease can also mask the presence of BA/CMPT and further complicate its management. The coexistence of LIP and BA/CMPT is rare, and to our knowledge, this is the only case reported in the literature. The pathogenesis of this coexistence is not yet well understood. Collaborative efforts and prospective studies are needed to further refine our understanding and optimize clinical management strategies for CMPT.

## AUTHOR CONTRIBUTIONS

Surgical and Medical Practices: Pinar Çağan, Feride Yaman, Özlem Yapıcıer, Cemal Asim Kutlu; Concept: Pinar Çağan, Feride Yaman, Ali Kimiaei, Seyedehtina Safaei, Houssam Eddine Youcefi, Alara Abu Saadeh, Özlem Yapıcıer, Cemal Asim Kutlu; Design: Ali Kimiaei, Seyedehtina Safaei, Houssam Eddine Youcefi, Alara Abu Saadeh; Data Collection or Processing: Ali Kimiaei, Seyedehtina Safaei, Houssam Eddine Youcefi, Alara Abu Saadeh, Özlem Yapıcıer; Analysis or Interpretation: Pinar Çağan, Feride Yaman, Ali Kimiaei, Seyedehtina Safaei, Houssam Eddine Youcefi, Alara Abu Saadeh, Özlem Yapıcıer, Cemal Asim Kutlu; Literature Search: Ali Kimiaei, Seyedehtina Safaei, Houssam Eddine Youcefi, Alara Abu Saadeh; Writing: Pinar Çağan, Feride Yaman, Ali Kimiaei, Seyedehtina Safaei, Houssam Eddine Youcefi, Alara Abu Saadeh, Özlem Yapıcıer, Cemal Asim Kutlu

## FUNDING INFORMATION

This research received no specific grant from any funding agency in the public, commercial, or not‐for‐profit sectors.

## CONFLICT OF INTEREST STATEMENT

The authors declare no potential conflicts of interest with respect to the research, authorship, and/or publication of this article.

## PATIENT CONSENT STATEMENT

Consent to participate in the study was obtained from the patient involved in the study. Documentation of informed consent is available for review by the Editor of this journal upon request.

## Data Availability

The datasets generated and/or analyzed during the current study are available from the corresponding author on reasonable request.
